# P067. Multimodal therapy in the management of MOH: a 3-year experience

**DOI:** 10.1186/1129-2377-16-S1-A135

**Published:** 2015-09-28

**Authors:** Valerio De Angelis, Francesca Cherubini, Gaia Nigrelli, Denise Erbuto, Paolo Martelletti

**Affiliations:** Department of Clinical and Molecular Medicine, Sapienza University of Rome, Rome, Italy; Department of Neurosciences, Mental Health and Sensory Organs, Suicide Prevention Center, Sant'Andrea Hospital, Sapienza University of Rome, Rome, Italy

The relationship between migraine and psychopathology has been clinically discussed in various studies. Medication-overuse headache (MOH) has been often found comorbid with emotional disturbances and disordered personality traits [1, 2]. This might play a role in the evolution of migraine to MOH and might be associated with higher risks of chronicization and/or relapses in drug abuse. Psychological disturbances may also be risk factors for a later development of MOH [3]. Since 2012 the Psychology Service of Sant'Andrea Regional Referral Headache Centre has offered short cycles of psychological interviews mostly oriented towards the support of MOH patients during the post-rehabilitation infusional phase in order to prevent relapses.

In these past 3 years 106 MOH patients afferent to the Headache Centre underwent the infusional therapy and were eligible for psychological support. Patients’ profiles highlighted avoidant and dependent personality traits together with a high level of anxiety and depressive mood. Patients showed a low perception of their personal skill, resources and a perception of their body mostly related to the head pain. The psychological intervention has been cognitive-behavioral and has improved patients’ perception of self relative to their body, their personal skills and resources.

Psychological support together with prophylaxis therapy with OnabotulinumtoxinA produced in patients a reduction of relapses in drug abuse of 38% compared to a sample of 108 patients age and gender matched that was not eligible for psychological support but received the detoxification and prophylaxis therapy.

This means that in some selected cases a multimodal therapy, consisting in a program of pharmacotherapy and psychological support, is necessary for the treatment of MOH patients in order to reduce the risks of relapses into drug abuse.

Written informed consent to publish was obtained from the patient(s).
